# Biospectroscopy Combined with Multivariate Analysis as Tools for Identifying *Trypanosoma cruzi* Discrete Typing Units in *Triatoma brasiliensis* (Hemiptera: Reduviidae: Triatominae)

**DOI:** 10.1021/acsomega.5c08763

**Published:** 2025-12-15

**Authors:** Jéssica T. Jales, Lavínia H. S. Pereira, Leomir A. S. de Lima, Raniery de O. Santana, Anne B. F. Câmara, Pedro Ramon da S. Aquino, Paulo Marcos M. Guedes, Andressa Noronha Barbosa-Silva Carvalho, Kássio M. G. Lima, Renata A. Gama, Antonia C. J. Câmara

**Affiliations:** 1 Graduate Program in Parasitic Biology, 28123 Federal University of Rio Grande do Norte, Natal 59072-970, Brazil; 2 Biological Chemistry and Chemometrics, Institute of Chemistry, 28123 Federal University of Rio Grande do Norte, Natal, RN 5072-970, Brazil; 3 Graduate Program in Pharmaceutical Sciences, 28123 Federal University of Rio Grande do Norte, Natal 59072-970, Brazil

## Abstract

Epidemiological surveillance of Chagas disease with determination of *Trypanosoma cruzi* (*T. cruzi*) positivity and genotyping is important for the adoption of control measures. The association of biospectroscopy with chemometric models can be used as an alternative tool to determine *T. cruzi* positivity and genotyping directly on the insect, with laser incidence, without damage to the insects, and without parasite isolation and growing. In this study, infrared spectroscopy (ATR-FTIR) combined with classification and authentication models was used for the identification of the experimental infection of *Triatoma brasiliensis* (*T. brasiliensis*) by different discrete typing units of *T. cruzi*. *T. brasiliensis* fourth and fifth instars were experimentally infected with 40,000 parasites (TcI, TcII, TcIII, or mixed infection) per milliliter of blood, and 1, 15, and 30 days after infection, infrared spectra were collected from the abdomen of each insect. The classification models, genetic algorithm linear discriminant analysis (GA-LDA) and successive projections algorithm linear discriminant analysis, achieved 100% sensitivity and specificity for TcII, the mixed infection, and control across all infection periods. For TcI and TCII, GA-LDA performed best in 15 days with 75% sensitivity and 94% specificity. The data-driven soft independent modeling of the class analogy model correctly classified most infected samples within the limits of both the training set and the test set, excluding the uninfected samples of the acceptance region, and achieving sensitivity and specificity close to 100%. Spectral differences, primarily attributed to proteins (amide III band, 1247–1307 cm^–1^) and nucleic acids (phosphate stretching vibrations, 1048–1085 cm^–1^), allowed for consistent discrimination between infected and uninfected insects. The polymerase chain reaction of kDNA analysis confirmed 92.5% (37/40) of infections in all triatomines submitted to experimental infection. Thus, the integration of ATR-FTIR, classification tools, and authentication models has been shown to be a rapid, noninvasive, and promising approach for the diagnosis and entomological surveillance of Chagas disease.

## Graphical abstract

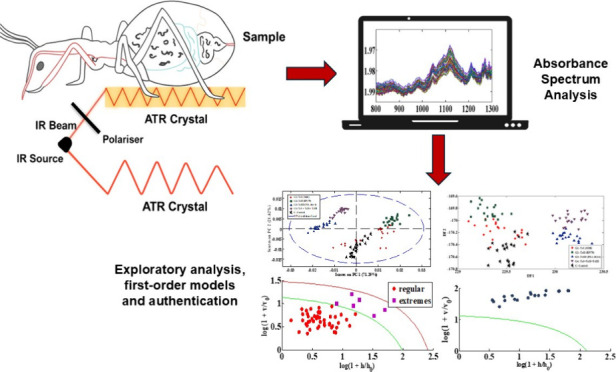

## 1 Introduction

*Trypanosoma cruzi* (*T. cruzi*) infection, also known as American Trypanosomiasis or Chagas disease, has been described by the World Health Organization as one of the main neglected tropical diseases in Latin America.^1,2^ It is estimated that 5.7 million individuals in 21 endemic countries of the Latin American Continent are infected with this parasite, and in Brazil, the estimate is that 1.2 million people are infected.^1^ The geographic distribution of this infection is mainly due to the circulation of 160 species of the insect vector, the triatomines (Hemiptera: Reduviidae: Triatominae), from which 70 are naturally infected with *T. cruzi*.^3–6^ These insects are characterized by hematophagy and a high degree of adaptation to domicile and peridomicile environments.^3,7,8^ Epidemiological surveillance with triatomine species studies, determination of *T. cruzi* positivity, and molecular characterization of the parasite are important tools for understanding parasite transmission mechanisms and controlling Chagas disease.

*T. cruzi* presents considerable biological heterogeneity regarding its morphology, DNA content, virulence, pathogenicity, and susceptibility to drugs^9,10^ and was classified into six discrete typing units (DTUs), also known as TcI–TcVI, based on different genetic markers.^11,12^ The characterization of *T. cruzi* isolates obtained from triatomines is complex and takes a long time, involving sterile isolation of the parasite by xenoculture,^13^ growth culture, obtaining parasite mass, DNA extraction, and performing DNA amplification techniques with at least three different genes. The protocol described by D’Avila et al.^14^ uses three genetic markers: divergent domain of rDNA (rRNA) gene 24Sα,^15^ mitochondrial gene cytochrome oxidase (COII) subunit II,^16^ and *T. cruzi* miniexon gene intergenic spacer (SL-IR).^17^ However, severe difficulties are encountered, such as sterile isolation of the parasite, difficulty in the growth of some isolates, possibility of DNA contamination, high cost to perform the techniques, and requirements of substantial training and a long period of analysis. Thus, evaluation of new methods for *T. cruzi* detection and genotyping in insects, fast to be performed, low cost, and less technical complexity are important for the routine use of these epidemiological data for the Chagas disease control.

Biospectroscopy through the use of Fourier transform infrared (FTIR) has allowed the identification of biomolecules,^18^ quantitative protein analysis,^19^ and identification and differentiation of viral infections,^20^ fungal infections,^21^ and bacterial infections.^22,23^ However, the application of this tool has not yet been reported in the identification of protozoa of medical importance. Spectroscopy has emerged as a cheaper and faster alternative for *T. cruzi* infection detection. Studies of compound behavior against radiation^20^ have been defined as a physical-chemical method based on the measurement of the vibration of a molecule excited by infrared radiation at a specific wavelength range.^22^ The range between 1800 and 900 cm^–1^ of the electromagnetic spectrum is known as biological fingerprint^24,25^ since it is in this range that information regarding biomolecules such as lipids (1750 cm^–1^), carbohydrates (1155 cm^–1^), proteins (amide I 1650 cm^–1^, amide II 1550 cm^–1^, and amide III 1260 cm^–1^), and DNA/RNA (1225 and 1080 cm^–1^) can be found.^24–26^ The Fourier transform infrared (FTIR) is an efficient technique for collecting and analyzing biological sample spectra.^20,24^

However, the application of the ATR-FTIR technique to different biological samples generates information-rich spectra, and the complexity of which can make direct data interpretation difficult, thus making the application of chemometric tools necessary for a better understanding of the spectra. In this way, the use of classification tools is highlighted as a powerful methodology in the discrimination of the *T. cruzi* identification.

In the specialized literature, some studies investigated the application of infrared spectroscopy for identifying contamination with *T. cruzi*.^27^ Near-infrared spectroscopy was applied for a rapid and noninvasive detection of *T. cruzi* in *Triatoma infestans* body parts in wet/dry excreta samples of the insect. An accuracy of 100% was obtained by using the partial least squares (PLS) model.^28^ The FTIR combined with PCA exploratory analysis was studied to differentiate among *L. chagasi*, *T. cruzi*, and *T. rangeli* species in the vibrational regions of polysaccharides, amide III, lipid esters, and fatty acids.^29^ Several machine learning techniques associated with ATR-FTIR were applied to detect the Chagas disease in blood serum, achieving an accuracy of 93%.

In addition to the exploratory analysis, the linear discriminant analysis (LDA) carries out segregation by maximizing the separation between classes through the ratio between the interclass variance and the intraclass variance.^30,31^ However, it is necessary to apply variable selection algorithms, such as the successive projections algorithm (SPA) and the genetic algorithm (GA), to reduce the dimensionality of the data by selecting only the most discriminating variables between classes. In addition, the use of authentication or one-class models can be applied to identify samples belonging to a specific class (target class) and distinguish them from all others, ensuring the authenticity of the samples.^32^ This combined approach aims to improve discriminatory capacity and reliability of the results, contributing to more robust analytical and diagnostic applications.

In this context, it becomes essential to associate spectroscopy with chemometric models capable of extracting, reducing, and interpreting relevant information. In this way, the determination of *T. cruzi* positivity and genotyping can be performed directly on the insect by biospectroscopy combined with a chemometrics method, in which this insect can be used for later identification based on its morphological characteristics. There is no need for parasite isolation and growth, as well as DNA extraction and amplification, which makes biospectroscopy a cheaper and faster method to perform than the traditional methodologies currently used. Thus, this work aims to evaluate the use of ATR-FTIR combined with classification and authentication chemometric models in the identification and genotyping of triatomine infection by *T. cruzi*.

## 2 Materials and Methods

### 2.1 Parasites and Feeding of Insects

Culture forms of *T. cruzi*, isolated from humans and triatomines, were previously characterized as TcI (3188), TcII (RN79), and TcIII (Pl 1.10.14)^33^ and cryopreserved in liquid nitrogen in the Laboratory of Parasites Biology and Chagas Disease (Department of Clinical Analyses and Toxicology, Health Sciences Center, Federal University of Rio Grande do Norte) (LABIOPAR/DACT/CCS/UFRN). Epimastigote forms were maintained in liver infusion tryptose (LIT media) with 10% bovine serum^34^ and later used in experimental triatomine infection.

Fifty (50) nymphs of fourth and fifth *Triatoma brasiliensis* (*T. brasiliensis*) stages were kindly provided by the LABIOPAR to conduct this research. The insects were separated into five (5) groups: negative control, consisting of insects fed with uninfected blood; G1 group, infected with TcI; G2 group, infected with TcII; G3 group, infected with TcIII; and G4 group, infected with blood containing TcI, TcII, and TcIII in the same proportion.

In experimental insect infection, 10 specimens in each group of *T. brasiliensis* nymphs were infected with 40,000 *T. cruzi* epimastigotes. The parasites were added to blood, collected from uninfected volunteers, and placed in the artificial feeder.^35^ Triatomines were fed for 30 min as described by Romaña (1947).^36^ Only well-fed insects were kept, and they were kept in the insectarium until 30 days after infection.

### 2.2 Positivity of Experimental Triatomine Infection

The positivity was verified after 1, 15, and 30 days of infection by spectroscopy, with acquisition of the infrared spectra, preprocessing, and analysis of the dataset. On the 30th day, the molecular analysis of the kinetoplast DNA of the parasite (kDNA) was also performed, following the protocol of Gomes et al. (1998).^37^ The same insects were used for spectroscopy and PCR kDNA analysis.

### 2.3 Spectral Acquisition with an ATR-FTIR Spectrophotometer

Spectra were collected from the midgut and rectal ampulla regions of each triatomine at 1, 15, and 30 days after experimental infection. For spectral acquisition, the live insects were placed with a straight-tipped clamp on the crystal inside the spectrophotometer (SHIMADZU, model IRAffinity-1, Japan), equipped with the ATR accessory. The spectra were obtained in the range of 4000–700 cm^–1^, with a spectral resolution of 4 cm^–1^, with 32 scans and 2 s measurement time per spectrum. The ambient temperature was approximately 22 °C. A background spectrum of air is the control spectrum since the insects remained in contact with the air during the procedure.

### 2.4 Preprocessing and Analysis of the Dataset

The data analysis was performed in MATLAB R2014a (MathWorks, Inc., USA) with PLS toolbox version 7.9.3 (Eigenvector Research, Inc., USA), the Classification toolbox (version 7.0) by Milano chemometrics and QSAR research group,^38^ and homemade routines. The raw spectra were preprocessed for the removal of external interferents. Each spectrum was cut in the range of 1300–800 cm^–1^ and received baseline (Automatic Weighted Least Squares) and smoothing Savitzky–Golay (15 points, second-order polynomial) adjustments. After preprocessing the dataset, followed by the exploratory data analysis, the principal component analysis (PCA) was performed for the evaluation of insect infection. The reduction of the number of variables by the PCA application was achieved by decomposing the original matrix X into its matrix products in scores and loadings, as shown in eq 1:

$$X=TP^{T}+E$$ 1

where *X* is the matrix *I* × *J* (*I* is the number of objects and *J* is the number of variables), *T* is the matrix of vector scores *t*_a_ *I* × *A* (*A* is the number of computed components), *P* is the matrix of vector loadings *J* × *A* (the superscript *T* indicates the transposed matrix *P*), and *E* is the residual matrix *I* × *J* kDNA extraction.^20^ For the classification models (successive projections algorithm linear discriminant analysis (SPA-LDA) and GA-LDA), the samples were divided into training (70%) and test (30%) subsets by applying the Kennard–Stone (KS) sampling algorithm.^39^ Training samples were used in the modeling procedure (including variable selection for LDA), while the prediction set was used only in the final classification evaluation. The variables selected by SPA and GA are based on minimizing the cost function *G* according to eq 2:

$$G=\frac{1}{N_{v}}\sum_{n=1}^{N_{v}}g_{n}$$ 2

where *N*_v_ represents the number of validation samples and *g*_*n*_ can be calculated according to eq 3:

$$g_{n}=\frac{r^{2}(x_{n},m_{I(n)})}{\min_{I(m)I\neq (n)}\;r^{2}(x_{n},m_{I(n)})}$$ 3

where *r* ^2^(*x*_*n*_ *,m_I_*_(*n*)_) is the squared Mahalanobis distance between the object *x*_*n*_ and the center of its true category, while *r* ^2^(*x*_*n*_ *,m_I_*_(*m*)_) represents the squared Mahalanobis distance between the object *x*_*n*_ and the center of the closest wrong category *m_I_*_(*m*)_.

After 30 days of experimental infection, the insects were examined individually according to the protocol of Barbosa-Silva et al. (2016).^7^ The intestinal contents were dissolved in saline solution, and an aliquot was used to observe the mobile parasites in direct examination. Another part was used for DNA extraction. DNA extraction was performed according to the protocol of Gomes et al. (1998).^37^ Polymerase chain reaction was used to detect the constant region of the kDNA minicircles (PCR kDNA), amplifying a 330 bp (base pair) fragment with the primers: 121 (5′-AAATAATGTACGGG (G/T) GAGATGCATGA-3′) and 122 (5′-GGT 33 TCGATTGGGGTTGGTGTAATATA-3′), as used by Degrave et al. (1988) and protocol by Gomes et al. (1998).^37^

The data-driven soft independent modeling of class analogy (DD-SIMCA) is based on principal component analysis (PCA) decomposition of the preprocessed data (eq 1).^40^ Based on the PCA results, the score distance (SD), *h*_i_, and the orthogonal distance (OD), ν_i_, are calculated. The SD is defined as the squared Mahalanobis distance and reflects how far the projection of sample i lies from the origin of the principal component (PC) space. In addition, the OD is defined as the squared Euclidean distance between sample i and the score subspace; that is, it represents how far the original data point is from its corresponding projection in the PC space.

The acceptance area (or decision threshold) for the target class is defined based on a predefined type I error rate, α. The acceptance conditions are given by

$$c\leq c_{\mathrm{crit}}(\alpha )$$ 4

where

$$c_{\mathrm{crit}}=X^{2}(1-\alpha ,N_{h}+N_{v})$$ 5

*X* ^2^ is the (1 – α) quartile of the chi-squared distribution with *N*_h_ + *N*_v_ degrees of freedom.^32,40^ After this step, the model is finalized and is ready for the classification of new data samples.

The dataset and the scripts applied in this study are depicted in the Supporting Information.

### 2.5 Model Quality Evaluation

In this study, accuracy measures such as sensitivity and specificity were applied to assess the test performance. Sensitivity corresponds to the probability of the test yielding a positive result when the disease is present, while specificity represents the probability of the test yielding a negative result in the absence of the disease. Both metrics range from 0 to 1 and are used to calculate quality parameters in the predictive model analysis.^41^

$$\mathrm{sensibility}\;(\%)=\frac{\mathrm{TP}}{\mathrm{TP}+\mathrm{FN}}\times 100$$ 6

$$\mathrm{sensibility}\;(\%)=\frac{\mathrm{TN}}{\mathrm{TN}+\mathrm{FP}}\times 100$$ 7

where FN is characterized by being the false negative and FP the false positive. TP is defined as true positive and TN as true negative.

## 3 Results and Discussion

### 3.1 Biospectroscopy Differentiates Infections of TcI, TcII, TcIII, and Mixed *T. cruzi* Infections in Triatomines

In this study, the discrete detection of *T. cruzi* in *T. brasiliensis* represents a significant clinical challenge since the characterization of *T. cruzi* isolates from triatomines is a complex and time-consuming process that requires sterile isolation of the parasite through xenoculture.^13^ Therefore, the need for a more complete diagnosis requires the development of methods that enable a more accurate identification of the infection caused by this insect. In this context, the use of spectroscopic techniques, such as attenuated total reflectance infrared spectroscopy (ATR-FTIR), combined with chemometric tools appears to be an alternative for this identification.

Figure 1A,C,E shows the raw ATR-FTIR spectra obtained in the 800–1300 cm^–1^ fingerprint region, corresponding to four *T. cruzi* infected groups and an uninfected control group. A high degree of band overlap is observed, which makes visual distinction between classes difficult and highlights the need for preprocessing the spectra, as illustrated in Figure 1B,D,F. In this way, the spectra were preprocessed using the Savitsky–Golay filter (15-point window, second-order polynomial) to smooth the signal, reduce high-frequency noise, and correct for small background variations.^42^ Baseline correction using the automatic weighted least squares method (second-order polynomial) was then applied to eliminate baseline slopes and curvatures, often caused by instrumental factors, sample variation, or light scattering, ensuring greater comparability between spectra. Baseline correction algorithms can minimize the effect of scattering artifacts in infrared spectroscopy datasets.^42^ Preprocessing in ATR-FTIR spectroscopy becomes necessary to improve data quality prior to chemometric analysis, reducing noise and unwanted effects that can obscure relevant chemical information.

**Figure 1:**
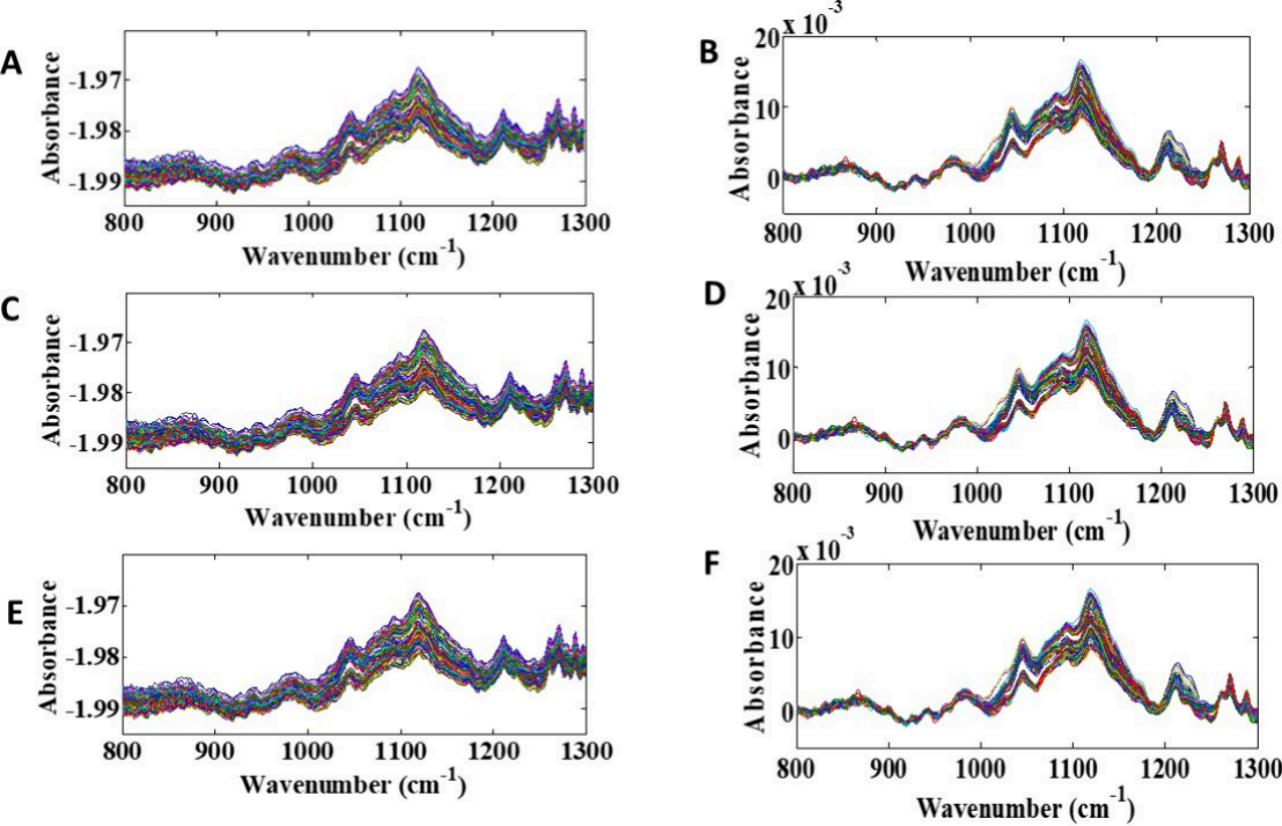
Original ATR-FTIR spectra of four *T. cruzi*-infected strains and one uninfected strain were analyzed. The raw spectra are shown in panels (A), (C), and (E), while the corresponding preprocessed spectra are shown in panels (B), (D), and (F), corresponding to 1, 15, and 30° days postinfection, respectively.

In addition, the spectral range of 800–1300 cm^–1^ is possible to observe bands corresponding to C–O and C–O–C stretching characteristic of polysaccharides. The feature in 1080–1100 cm^–1^ is correspondent to the vibrations of phosphates (PO_2_^–^) of nucleic acids. Between 1230 and 1250 cm^–1^, the presence of asymmetric phosphate stretching (PO_2_^–^) of nucleic acids and phospholipids is noted, and the region of 1247–1307 cm^–1^ correspond to the amide III band, which is associated with proteins.^43^ However, even after applying preprocessing, it is possible to notice that the bands continue to overlap. Therefore, it is necessary to associate these spectroscopic data with chemometric algorithms for the correct classification of mosquitoes.

As shown in Figure 1, there is a high similarity between the ATR-FTIR spectra for the three studied days, indicating a spectral overlapping from the groups when plotted together, not being possible to distinguish between then. In this way, it is necessary to use techniques capable of differentiating between the classes by maximizing the differences between them, such as the multivariate classification algorithms and one-class models.

### 3.2 Principal Component Analysis (PCA)

The application of principal component analysis (PCA) to the spectra obtained by ATR-FTIR resulted in the formation of five distinct groups corresponding to the classes: G1 (TcI), G2 (TcII), G3 (TcIII), G4 (TcI + TcII + TcIII), and C (control—not infected), as shown in Figure 2. The model presented a cumulative variance of 95%, demonstrating consistent separation between classes across the three infection periods evaluated. Principal component 1 (PC1) concentrated the largest portion of the data variability, providing satisfactory separation between groups, with no overlap between most classes. The blue dashed ellipse, representing the 95% confidence interval, encompassed all samples, indicating the absence of outliers or atypical samples. The separation between classes was clear, except for TcI, which was closer to the not infected group and TcII samples across the three infection periods, possibly due to the inherent characteristics of the first type of infection. The remaining groups formed more homogeneous clusters, suggesting more uniform spectral profiles. These results reinforce the need to apply chemometric tools to the spectral data, enabling their application in unknown samples and contributing to more accurate clinical diagnoses in the future.

**Figure 2:**
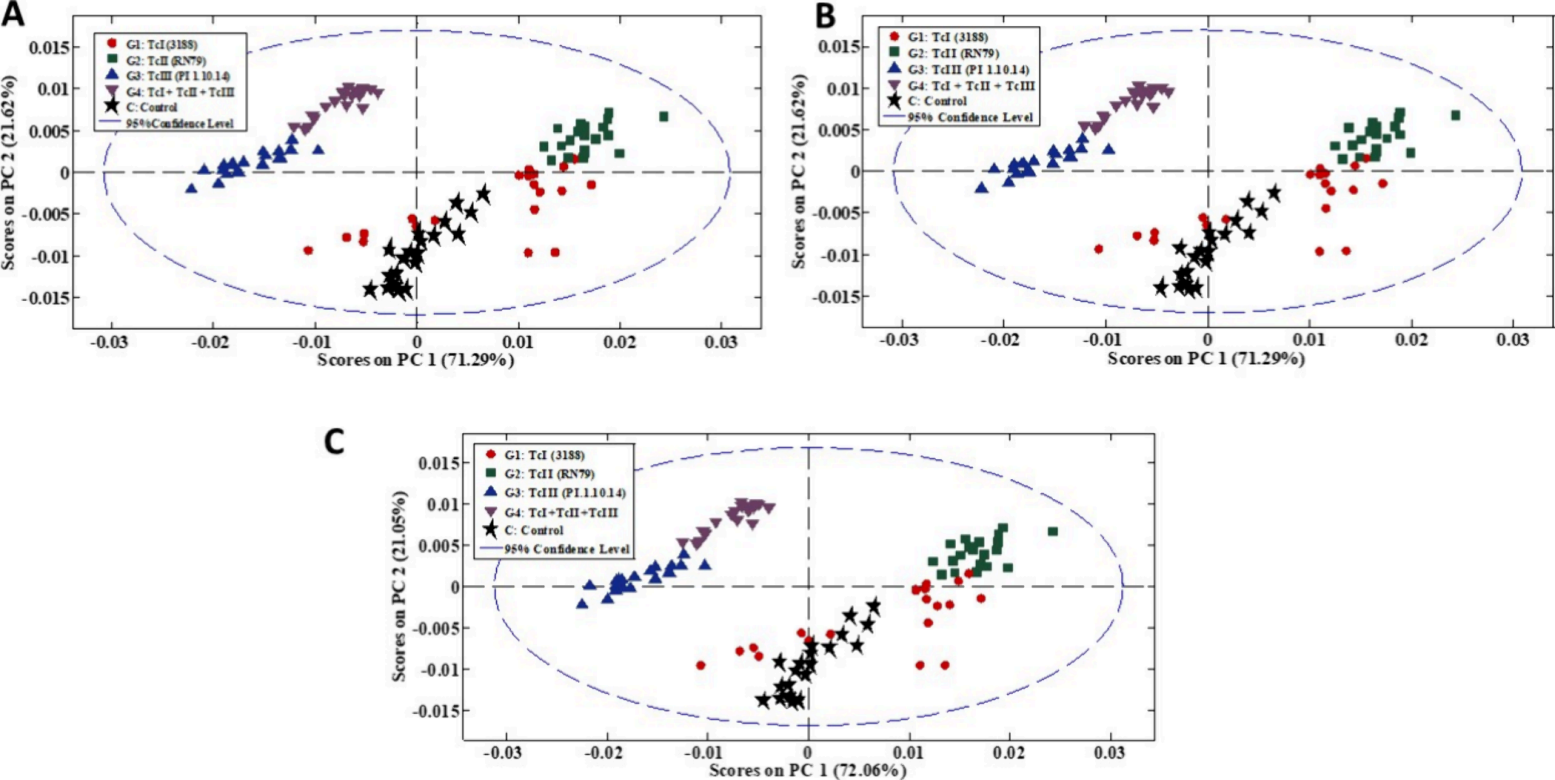
PCA score graphs for the three infection periods: 1st day (A), 15th day (B), and 30th day (C), including the four infected classes and the uninfected control class.

The PCA loadings for the 3 days give similar spectra for each calculated PC; for this reason, Figure 3 depicts the loadings for the determination of *T. cruzi* in 15th day. The analysis of PCA loadings was performed by observing the most prominent peaks in the spectrum. In this way, the peaks that presented a higher absorbance made greater contributions to the analysis of the information in that principal component. Each absorbance represents a compound with a wavelength. Analyzing the loading graph dataset, the most significant wavelengths for class separation were for PC1 and PC2, between 1500 and 1600 cm^–1^, 1650 cm^–1^, and between 1700 and 1800 cm^–1^, which correspond to amide II, amide I, and lipids, respectively. For PC3, between 1000 and 1100 cm^–1^, between 1450 and 1500 cm^–1^, and between 1700 and 1800 cm^–1^ correspond to nucleic acids, proteins (COO−), and amides, respectively.

**Figure 3:**
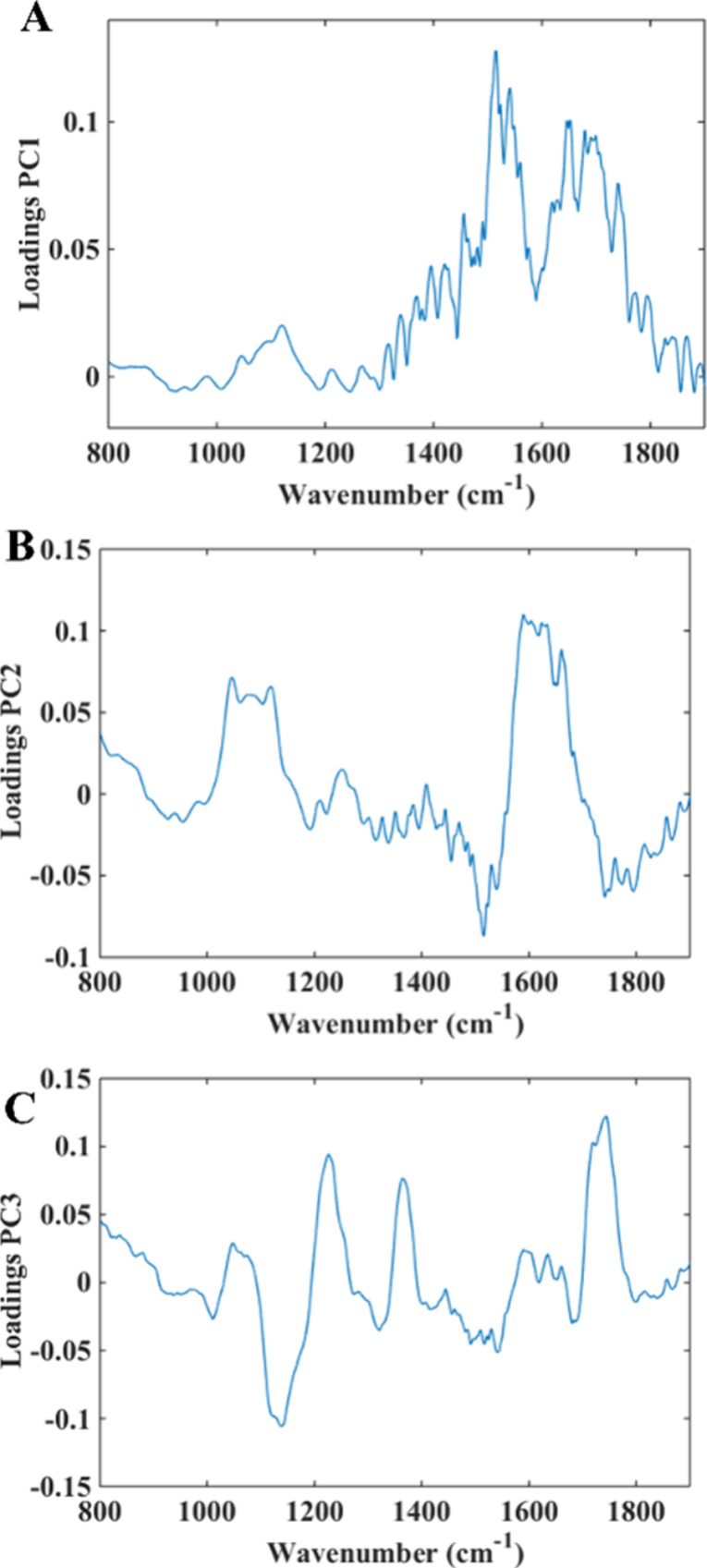
PCA loading graphs for the three principal components on day 15: PC1 (A), PC2 (B), and PC3 (C).

### 3.3 SPA-LDA and GA-LDA Models

Variable selection techniques such as the genetic algorithm (GA) and successive projection algorithm (SPA) combined with linear discriminant analysis (LDA) can be applied to spectral data to develop a model capable of discriminating between contaminated and uncontaminated *T. cruzi* samples in *T. brasiliensis* samples.

The cost function *G* (eq 2) was used to evaluate the efficiency of variable selection procedures in the classification models. As defined in eq 3, the parameter *g_n_* reflects the relative separation of each validation sample from its true class compared to the closest alternative class. In our analysis, lower values of *G* indicated an improved discrimination capacity of the model, confirming the adequacy of the selected variables. Both SPA and GA successfully minimized *G*, which resulted in an enhanced classification performance of the LDA models. These findings demonstrate that the variable selection strategies based on eqs 2 and 3 effectively improved the robustness of the spectroscopic classification, in agreement with previous reports on chemometric modeling.^27^ The model’s performance was evaluated using the sensitivity and specificity results, as shown in Table 1.

**Table 1:** Results of the Figures of Merit for the SPA-LDA and GA-LDA Models in the Periods of 1st, 15th, and 30th Days of Infection

			**cross validation**		**prediction**	
**infection time**	**model**	**class**	**sensitivity (%)**	**specificity (%)**	**sensitivity (%)**	**specificity (%)**
day 1	SPA-LDA	G1—TcI	93%	100%	67%	100%
		G2—TcII	100%	98%	100%	100%
		G3—TcIII	98%	100%	100%	100%
		G4—TcI + TcII + TcIII	100%	99%	83%	100%
		C—control	100%	100%	100%	100%
	GA-LDA	G1—TcI	92%	100%	67%	100%
		G2—TcII	100%	98%	100%	100%
		G3—TcIII	97%	100%	100%	100%
		G4—TcI + TcII + TcIII	100%	99%	100%	100%
		C—control	100%	100%	100%	100%
day 15	SPA-LDA	G1—TcI	92%	100%	67%	100%
		G2—TcII	100%	98%	100%	92%
		G3—cIII	97%	100%	100%	75%
		G4—TcI + TcII + TcIII	100%	100%	100%	100%
		C—control	100%	100%	100%	100%
	GA-LDA	G1—TcI	99%	100%	83%	100%
		G2—TcII	100%	100%	100%	100%
		G3—TcIII	94%	98%	100%	100%
		G4—TcI+ TcII+ TcIII	93%	98%	100%	100%
		C—control	100%	100%	100%	100%
day 30	SPA-LDA	G1—TcI	92%	100%	67%	100%
		G2—TcII	100%	98%	100%	100%
		G3—TcIII	97%	100%	100%	100%
		G4—TcI + TcII + TcIII	100%	100%	100%	100%
		C—control	100%	100%	100%	100%
	GA-LDA	G1—TcI	94%	100%	67%	100%
		G2—TcII	100%	98%	100%	100%
		G3—TcIII	94%	98%	100%	100%
		G4—TcI + TcII + TcIII	94%	99%	100%	100%
		C—control	100%	100%	100%	100%

Table 1 presents the figures of merit of *the successive projections algorithm linear discriminant analysis* (SPA-LDA) and g*enetic algorithm linear discriminant analysis* (GA-LDA) models for the five sample classes, considering the different days of infection. On the first day, reduced sensitivity is observed for class TcI, which is possibly due to the initial stage of infection, in which the model does not perform satisfactorily in identifying this class. On the other hand, TcII obtained a specificity of 100%, indicating a good ability of the model to correctly distinguish samples that do not belong to this class. Classes TcIII, TcI + TcII + TcIII, and C also showed good sensitivity and specificity results when applying the SPA-LDA model. When compared with SPA-LDA, the GA-LDA model obtained similar results. On day 15 after infection, the sensitivity values increased for classes TcI, reaching 83%, demonstrating that GA-LDA can be considered the most efficient model. On day 30 of infection, both models maintained the previously observed pattern. These results indicate that from day 30 of infection onward, the GA-LDA model begins to demonstrate a more effective ability to identify classes TcI and TcII, reflecting an improvement in performance as the infection progresses.

Table 2 summarizes the confusion matrix for each model, providing a visual representation of the models’ performance for each class. In the test set, two samples of the TcI group were misclassified for almost all the models developed in this study, except for GA-LDA for day 15, where only one sample was misclassified. These results are in accordance with the sensitivity and specificity parameters depicted in Table 1.

**Table 2:** Results of the Confusion Matrices Obtained by the SPA-LDA and GA-LDA Models in the Classification of Triatomine Bugs Infected with Different Strains of *T. cruzi* after 1st, 15th, and 30th Days of Infection

	SPA-LDA					GA-LDA				
day 1	G1	G2	G3	G4	C	G1	G2	G3	G4	C
predicted as G1—TcI	4	2	0	0	0	4	2	0	0	0
predicted as G2—TcII	0	6	0	0	0	0	6	0	0	0
predicted as G3—TcIII	0	0	6	0	0	0	0	6	0	0
predicted as G4—TcI + TcII + TcIII	0	0	1	5	0	0	0	0	6	0
predicted as C—control	0	0	0	0	6	0	0	0	0	6

	SPA-LDA					GA-LDA				
day 15	G1	G2	G3	G4	C	G1	G2	G3	G4	C
predicted as G1—TcI	4	2	0	0	0	5	1	0	0	0
predicted as G2—TcII	0	6	0	0	0	0	6	0	0	0
predicted as G3—TcIII	0	0	6	0	0	0	0	6	0	0
predicted as G4—TcI + TcII + TcIII	0	0	0	6	0	0	0	0	6	0
predicted as C—control	0	0	0	0	6	0	0	0	0	6

	SPA-LDA					GA-LDA				
day 30	G1	G2	G3	G4	C	G1	G2	G3	G4	C
predicted as G1—TcI	4	2	0	0	0	4	2	0	0	0
predicted as G2—TcII	0	6	0	0	0	0	6	0	0	0
predicted as G3—TcIII	0	0	6	0	0	0	0	6	0	0
predicted as G4—TcI + TcII + TcIII	0	0	0	6	0	0	0	0	6	0
predicted as C—control	0	0	0	0	6	0	0	0	0	6

The lower sensitivity observed at day 1 may be related to the early stage of *T. cruzi* infection, when the parasite load is still low, and the biochemical alterations in the host or vector are not yet sufficiently pronounced to be detected by ATR-FTIR spectroscopy. At this stage, the spectral differences between infected and noninfected samples may be subtle, reducing the discriminatory power of the GA-LDA model. In contrast, by day 15, the infection is more established, leading to more evident biochemical changes, such as alterations in proteins, lipids, and nucleic acids, which are more easily captured by the vibrational spectra. This explains the improved sensitivity of GA-LDA current point. From a practical perspective, these results suggest that while ATR-FTIR combined with GA-LDA holds promise as a diagnostic or surveillance tool, its applicability for very early detection of infection may be limited, and complementary methods could be required for reliable identification in the initial stages.

Figure 4 shows the discriminant function plots generated by the SPA-LDA and GA-LDA models, applied to ATR-FTIR spectra, to differentiate samples contaminated and uncontaminated by *T. cruzi* at different infection times (1, 15, and 30 days). Graphs (A), (C), and (E) correspond to the results of the SPA-LDA model, while graphs (B), (D), and (F) represent the projections obtained by the GA-LDA model. In the three periods analyzed, the SPA-LDA model was able to efficiently separate the control group from the infected samples, as evidenced by the clear distribution of classes in the discriminant space. In addition to distinguishing between contaminated and uncontaminated samples, the model demonstrated effectiveness in differentiating among TcI (G1), TcII (G2), TcIII (G3), and the mixed group TcI + TcII + TcIII (G4). This discriminative capability reinforces the performance of the successive projection algorithm combined with linear discriminant analysis, suggesting its potential in the biochemical characterization of *T. cruzi* infections and in spectroscopy-based diagnostic applications. Similarly, the GA-LDA models demonstrated excellent performance across the three experimental time points, particularly in graph (F), corresponding to day 30 postinfections. At this point, the separation among the five classes was even more pronounced, especially for the control group (C), whose samples (black stars) clearly stand out from the infected ones. This highlights the high specificity of the GA-LDA model, reflecting its ability to capture unique spectral patterns from uninfected samples. Furthermore, the TcIII strains (G3) and the mixed strain TcI + TcII + TcIII (G4) remained well-clustered, with defined boundaries, even after infection for 30 days, indicating robustness in identifying these classes. Strains TcI (G1) and TcII (G2), although closer to each other, still showed sufficient distinctions, suggesting that the model is also sensitive to more subtle biochemical variations between these lineages. Taken together, the results obtained with both discriminant models demonstrate the effectiveness of chemometric approaches applied to ATR-FTIR spectral data in classifying samples associated with *T. cruzi* infection. The accuracy observed in the separations showed the potential of these tools to support differential diagnosis, even in the early or advanced stages of infection.

**Figure 4:**
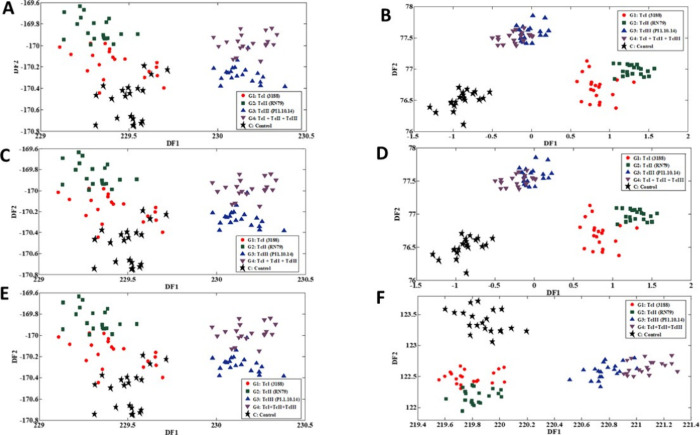
Projection of samples onto the discriminant functions generated by the SPA-LDA and GA-LDA models, applied to ATR-FTIR spectra to distinguish between *T. cruzi*-contaminated and -uncontaminated classes. Panels (A), (C), and (E) correspond to the results of the SPA-LDA model, while panels (B), (D), and (F) refer to the GA-LDA model. The analyses were performed with data collected after 1 day of infection (A, B), 15 days (C, D), and 30 days (E, F).

Furthermore, possible changes in the biochemical fingerprints of the classes were investigated by correlating them with the variables selected by each combined approach. The most relevant wavenumbers extracted by both the SPA-LDA and GA-LDA models are presented in Table 3.

**Table 3:** Wavenumbers Selected by the SPA-LDA and GA-LDA Models for the 1st, 15th, and 30th Day of Infection

**infection time**	**SPA-LDA (cm** ^**–1**^ **)**	**GA-LDA (cm** ^**–1**^ **)**
day 1	1.065, 1.119, 1.217, 816, 868, 1.018	885, 928, 1.101, 1.110, 1.171, 1.211, 1.231, 1.279
day 15	1.065, 1.119, 1.217, 816, 868, 1.018	885, 928, 1.101, 1.110, 1.171, 1.211, 1.231, 1.279
day 30	1.065, 1.119, 1217, 816, 868, 1018	1.203, 1.026, 1.030, 1.061, 1.076, 1.151, 1.204, 1.236

Table 3 summarizes the wavenumbers selected by the SPA-LDA and GA-LDA models for the three infection periods. The SPA-LDA model selected the same wavenumbers for days 1, 15, and 30, demonstrating recurring spectral patterns throughout disease progression. Among these wavenumbers, 1065 cm^–1^ stands out, which can be attributed to the C–O–R stretching characteristic of phosphodiester and ribose groups of nucleic acids in the absence of glycogen, possibly related to parasite proliferation or the host cellular response. The band at 1119 cm^–1^ corresponds to the symmetric P–O–C stretching and, possibly, to the C–O stretching, while the band observed at 1217 cm^–1^ is associated with the asymmetry of the PO_2_^–^ phosphate group (phosphate I); both may indicate alterations in membrane phospholipids, often associated with parasite–cell interactions. The feature in 1018 cm^–1^ is attributed to C–O stretching, C–C stretching, and O–C–H angular deformation, in addition to vibrations characteristic of aromatic rings or cyclic carbohydrate structures, which may reflect degradation or reorganization of polysaccharides in the extracellular matrix, a phenomenon linked to invasion and tissue remodeling caused by *T. cruzi*.^43^

The results obtained by the GA-LDA model reveal that *T. cruzi* infection induces chemical modifications detectable by ATR-FTIR from the early stages, showing an evolutionary pattern that reflects both parasite adaptation and the host’s biochemical response. In the first periods (1st and 15th day), the wavenumbers 1110 and 1171 cm^–1^ indicate alterations in structural polysaccharides, such as cellulose, and in glycomaterials, suggesting modifications in the host’s cellular matrix and carbohydrate metabolism. The bands at 1211 and 1231 cm^–1^, associated with phosphate groups (PO_2_^–^) and the amide III region (1247–1307 cm^–1^), as well as nucleic acid vibrations (1048–1085 cm^–1^), suggest changes in phosphorylation processes, membrane integrity, and parasite–cell interactions. The presence of the 1279 cm^–1^ band reinforces the involvement of structural proteins and polysaccharides in the initial response to infection.

On the 30th day, bands primarily related to carbohydrate metabolism and cellular protein content are observed. The 1025 and 1030 cm^–1^ bands indicate strong contributions from glycogen and simple carbohydrates (glucose and fructose), reflecting possible consumption or reorganization of these energy reserves due to persistent infection. The 1060 cm^–1^ band, characteristic of nucleic acids, suggests intense replication or degradation of genetic material, while the 1076 and 1151 cm^–1^ bands confirm changes in phosphate and glycogen. The 1204 and 1236 cm^–1^ bands indicate modifications in structural proteins, collagen, and nucleic acids, possibly associated with tissue remodeling and maintenance of the parasite’s life cycle in the host.

These spectral differences, attributed to proteins, nucleic acids, and phospholipids, may be correlated to fundamental biochemical processes occurring during *T. cruzi* infection. Variations in proteins (amide III, 1247–1307 cm^–1^) suggest alterations in host protein synthesis and degradation, reflecting potential mechanisms of metabolic reprogramming in infected tissues. The bands attributed to nucleic acids (1048–1085 cm^–1^) may be related to parasite replication and the consequent increase in DNA and RNA, especially in the early stages of infection, when multiplication is more intense. Finally, the alterations in phospholipids indicate changes in membrane composition and lipid metabolism, which are crucial for parasite survival and host–parasite interactions.

Thus, the identification of these spectral signatures reinforces the potential of spectroscopy associated with chemometrics (GA-LDA) as a diagnostic and disease-monitoring tool, capable of discriminating between different stages of infection, in agreement with previous biospectroscopy studies on parasitic infections.

The consistent selection of these wavelengths in the three periods evaluated indicates that the chemical changes detected are present from the initial to the most advanced stages of infection, functioning as spectral biomarkers for the diagnosis and monitoring of the disease.

### 3.4 DD-SIMCA

As an alternative to classical multivariate classification models, one-class models were applied in this study to identify contaminated species. Thus, the *data-driven soft independent modeling of class analogy* (DD-SIMCA) model was applied to classify samples belonging to a class of interest based on internal data variability. The training of this model was constructed using exclusive samples from the target class, comprising 56 samples contaminated with *T. cruzi* infection, distributed among the four classes. For validation purposes, 20 noninfected control class samples were used, corresponding to the 1st, 15th, and 30th days of infection. In addition, outliers detected in the samples were removed prior to the application of the DD-SIMCA model for each preprocessed dataset.

Graphs (panels) A–E of Figure 5 depict the training group (56 contaminated samples) across the three infection stages and reveal no significant outliers. One inconsistent sample was initially identified and excluded, after which the reconstructed plots displayed only regular or extreme samples within acceptable statistical limits. The decision boundaries are defined by two thresholds: the 95% confidence limit (green line), which separates regular (red) from extreme (purple) samples, and the 99% exclusion limit (red line), above which samples are considered true outliers and therefore rejected by the model. This distribution indicates that the DD-SIMCA model effectively characterizes the internal variability of the contaminated class while maintaining rigorous statistical control of potential anomalies.

**Figure 5:**
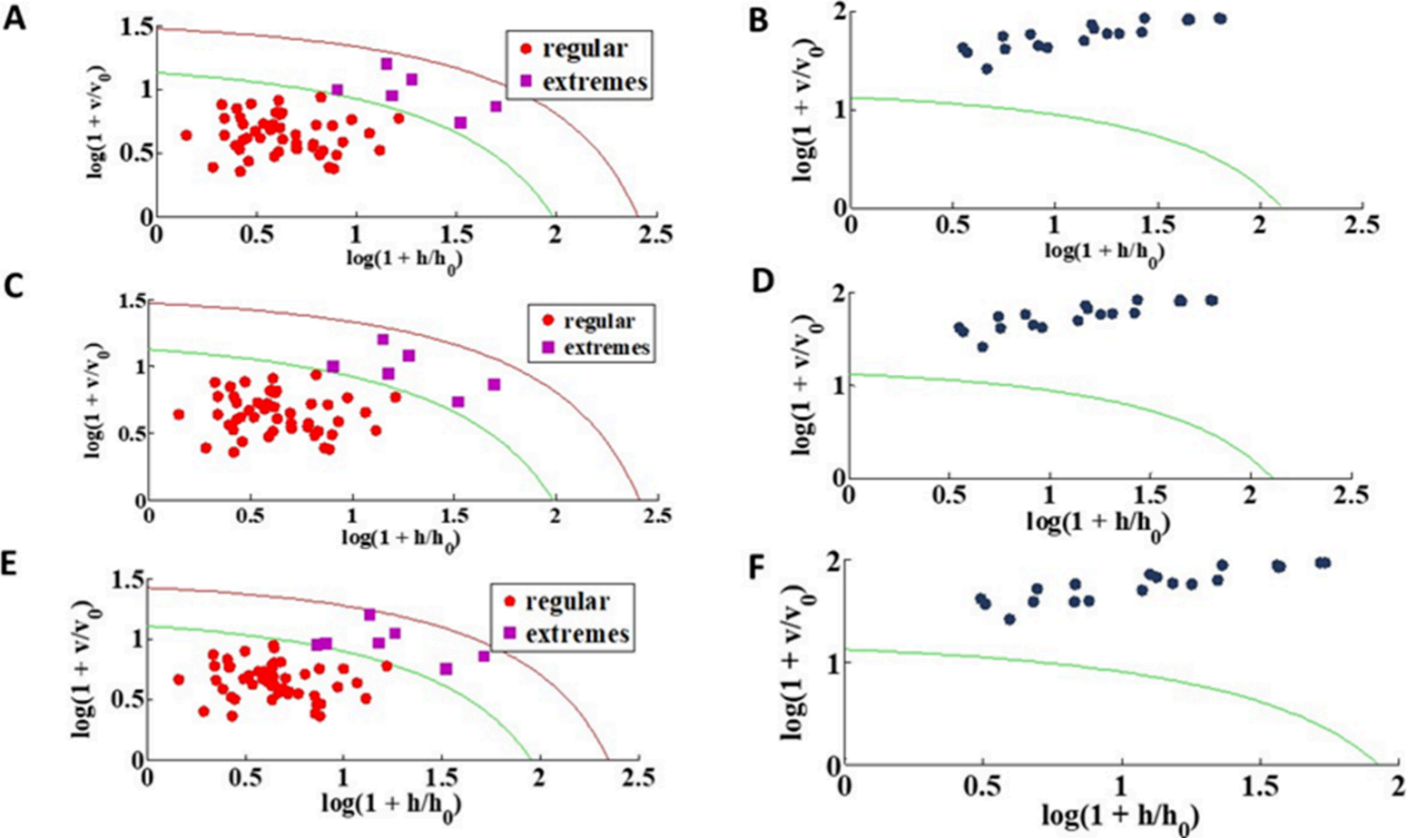
DD-SIMCA models applied for the training samples and predictions for the (A, B) 1st, (C, D) 15th, and (E < F) 30th day of infection. All models were built using 2 PCs and with a confidence level of 95% (α = 0.05).

On all 3 days analyzed (1st, 15th, and 30th), most samples in the training group remained within the confidence limit, demonstrating that the model was able to adequately capture the internal variability of the contaminated class. The samples classified as extreme (purple) exceeded the confidence limit but are still within the model’s acceptance limit and are not considered outliers. This indicates that these samples are statistically further from the class meaning but still consistent with the expected behavior.

This graphical analysis (Figure 5) provides more than statistical validation; it also reflects the biological consistency of the contaminated samples across different infection stages. The absence of significant outliers in the contaminated group suggests that the metabolic response to infection produces a homogeneous statistical signature that can be robustly modeled. The consistency of this pattern across days demonstrates the temporal robustness of DD-SIMCA, maintaining a stable performance even as the infection progressed.

Graphs B–F of Figure 4 illustrate the projection of the 20 control (uncontaminated) samples across the three infection stages. In every case, these samples (blue) plotted beyond the 95% confidence limit (green line) and were consistently rejected by the model as noncontaminated. This result confirms complete specificity, with no false acceptance, and demonstrates that the DD-SIMCA model reliably distinguishes the infected class from controls. Such performance highlights its capacity to capture infection-specific variability while minimizing the risk of misclassification, a key requirement for diagnostic and monitoring applications.

These results support the applicability of the DD-SIMCA model in contexts where multiclass supervised modeling is not feasible or desirable, evidencing its ability to detect significant deviations from the statistical behavior of the modeled class and its effectiveness in one-class classification.

Table 4 shows the results of the sensitivity and specificity obtained in the training and prediction sets for the models developed by using the DD-SIMCA algorithm. In the three distinct intervals of infection days evaluated (days 1, 15, and 30), both modeling environments presented equivalent performance, maintaining excellent discriminant ability throughout all stages. Under these conditions, the models achieved 90% sensitivity, demonstrating that most of the contaminated samples were correctly recognized as belonging to the target class. In the prediction set, performed with 20 samples, the models achieved 100% sensitivity and specificity, demonstrating perfect detection and rejection, with no occurrence of false acceptances or rejections.

**Table 4:** Results of the Figures of Merit (Sensitivity and Specificity) Obtained for the Training Set and Prediction for the Models Developed through DD-SIMCA on the Different Days of Infection

DD-SIMCA	**figure of merit**
TRAIN	90% (SENS.)
PREDICT	100% (SENS. AND SPEC.)

These results confirm the high effectiveness of DD-SIMCA in one-class scenarios, ensuring excellent performance in distinguishing between infected and noninfected samples, regardless of the implementation environment.

It should be noted that our findings are exclusively based on laboratory experiments under a controlled system. The field application of ATR-FTIR most likely requires fresh calibration models to account for environmental variations associated with triatomines: temperature, humidity, and contamination by dust or biological debris. The difficulty of standardizing the preparation and positioning of insects on the ATR crystal, as well as the physiological state and integrity of the vectors, can introduce spectral noise or systematic variations that might affect data quality.^44^ Furthermore, improper handling of triatomines, especially under uncontrolled conditions, can lead to degradation of the biological material or the presence of chemical interferents that mask the vibrational signatures associated with the parasite’s different discrete typing units (DTUs).^28^ In addition, each triatomine species has its unique infrared spectral characteristics, so a universal model incorporating multiple species could therefore be developed for predicting infection in those species. Alternatively, species specific models could be developed for heterogeneous settings.^27^

### 3.5 Biospectroscopy Presents Higher Sensitivity than PCR for *T. cruzi* Detection

The kDNA positivity for identification of the 330 bp fragment was 92.5% (37/40) in the triatomines submitted to experimental infection. Figure 6 shows the polyacrylamide gel representative of kDNA PCR of the TcI, TcII, and TcIII infected groups and mixed infection with the three DTUs, revealing the 330 bp band in the majority of the samples.

**Figure 6:**
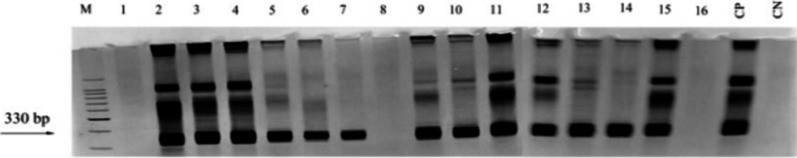
2% polyacrylamide gel representative of the positivity of intestinal suspensions of *T. brasiliensis* infected with Tc I, TcII, and/or TcIII. Samples 1 and 2 represent the group infected with TcI; samples 3–6, TcII infected group; samples 7–13, infected with TcIII; and samples 14–16, mixed infections. CP = positive control; CL BRENER. CN = Control: uninfected insects, M = molecular size marked 100 pb, BP = base pairs.

Samples 1 (TcI), 8 (TcIII), and 16 (mixed infection) did not amplify the 330 bp band. Considering the total of 10 samples per group, 90% (9/10) of the samples from the group infected with TcI and TcIII and submitted to mixed infection were positive for kDNA, while 100% (10/10) of the insects infected only by TcII were positive for kDNA.

The identification of *T. cruzi* infection by kDNA was considered by several authors as the technique that presents the highest percentage of identification, with this investigation varying according to the triatomine species, technique, and the origin of the insects, natural or experimental infection.^8,45–47,48^ kDNA analysis showed that 90% of infections by TcI, TcIII, and mixed infections were identified; on the other hand, 100% of TcII infections were identified. Thus, kDNA showed a sensitivity of 92.5% for the identification of infections in *T. brasiliensis*, a percentage higher than that found in ref^37^, whose percentage of positivity for kDNA was approximately 24% in natural infection. This difference may have occurred due to the higher quantity of protozoa used in experimental infections, increasing the positivity of the technique. The application of medium infrared biospectroscopy proved to be promising for the identification of *T. brasiliensis* infection by *T. cruzi*. Due to its ease execution and low cost, this methodology can be applied in the entomological surveillance procedures of this infection, aiding in a fast, simple, and reliable way. This work standardized the classification model necessary to evaluate the methodology as an auxiliary tool in the diagnosis of *T. cruzi* infection. However, validation procedures are necessary so that they can be used as a diagnostic method. The use of medium infrared showed robust results in this work, but other spectrum bands can be applied in the identification of *T. cruzi* infection in vector insects, such as near-infrared (NIR). Although ATR-FTIR spectroscopy provides rapid, reagent-free, and nondestructive analysis, its application in field settings may face practical challenges. The high cost and limited portability of conventional FTIR instruments, as well as the need for specific operator training, may restrict its immediate applicability for large-scale surveillance of *T. cruzi* infection. However, recent developments in compact and portable FTIR devices, combined with simplified sample-handling protocols, indicate that the use of ATR-FTIR in point-of-care or field-based scenarios is becoming increasingly feasible. In this context, the integration of standardized workflows and minimal operator training could enable ATR-FTIR to complement traditional diagnostic methods, especially in regions where rapid screening is essential.

## 4 Conclusions

In this study, the potential of infrared spectroscopy is combined with classification and authentication models for the identification of the infection of *T. brasiliensis* with *T. cruzi*, obtaining highly effective results and allowing the discrimination of DTUs classified as TcI, TcII, TcIII, and mixed infections. This differentiation can be assessed through the differential detection of protein and nucleic acid spectral signatures. The multivariate classification models demonstrated high discriminatory power: GA-LDA and SPA-LDA achieved 100% sensitivity and specificity for the TcII, mixed infections, and control groups across all infection periods. For TcI and TcII, GA-LDA achieved its best performance at 15 days postinfection, with 75% sensitivity and 94% specificity, while SPA-LDA also showed high and consistent values, varying slightly with DTU and time postinfection. The DD-SIMCA-based authentication models correctly classified most infected samples within the model limits during training, while uninfected samples in the test sets were correctly identified as out of the model, and infected samples remained within the acceptance limits, resulting in sensitivity and specificity values close to 100% across all sets and all infection intervals. These results confirm that the integration of ATR-FTIR and chemometric approaches constitutes a fast, efficient, simple, and low-cost tool for the identification of a disease vector whose accurate detection is still complex, laborious, and restricted to specialized research laboratories. The findings reported here have an important application in the field of entomological surveillance, prevention, and control of Chagas disease vectors. Furthermore, the perspectives of research using infrared spectroscopy combined with chemometric tools for the detection of infections of *T. brasiliensis* with *T. cruzi*, including in situ analysis, are vast, highlighting the potential of this methodology.

## Supplementary Material



## Notes

Dataset and scripts applied (DOCX)
