# Supplementary material for: Biospectroscopy Combined with Multivariate Analysis as Tools for Identifying Trypanosoma cruzi Discrete Typing Units in Triatoma brasiliensis (Hemiptera: Reduviidae: Triatominae)
Source: ACS Omega. 2025 Dec 15;10(51):62938–49. doi: 10.1021/acsomega.5c08763 (PMC12756755; doi:10.1021/acsomega.5c08763)
Supplement: Supplementary file 1 [file ao5c08763_si_001.docx]

**Supporting Information**

**Biospectroscopy combined with multivariate analysis as tools for identifying *Trypanosoma cruzi* discrete typing units in *Triatoma brasiliensis* (Hemiptera: Reduviidae: Triatominae)**

Jéssica T. Jales^1^, Lavínia H. S. Pereira^2^, Leomir A. S. de Lima^2^, Raniery de O. Santana^3^, Anne B. F. Câmara^2^, Pedro Ramon da S. Aquino^3^, Paulo Marcos M. Guedes^1^, Andressa Noronha Barbosa-Silva Carvalho^1^, Kássio M. G. de Lima^2,*^, Renata A. Gama^1^, Antonia Claudia J. da Câmara^1^

^1^Graduate Program in Parasitic Biology, Federal University of Rio Grande do Norte, Natal, Brazil.

^2^Biological Chemistry and Chemometrics, Institute of Chemistry, Federal University of Rio Grande do Norte, Natal, RN, 5072-970, Brazil.

^3^Graduate Program in Pharmaceutical Sciences, Federal University of Rio Grande do Norte, Natal, Brazil.

^*^E-mail: [kassio.lima@ufrn.com](mailto:kassio.lima@ufrn.com)

1 – Script

%%Abrir Espectro Barbeiro

[ WN, WL, DS ] = abrirEspectroFULL()

%%Transfotmar WL em comprimento de onda

nm = WL_S_Coccus(1,:)*10^9;

%%Plotar os espectros

plot(cm, G1)

%% Transformar de transmitancia para absorbancia

G1_ABS=-log10(G1);

G2_ABS=-log10(G2);

G3_ABS=-log10(G3);

G4_ABS=-log10(G4);

C_ABS=-log10(C);

%% Transpor a matriz cm e cmcut

Transposta = transpose(cm);

Transposta2 = transpose(cmcut);

%Corte da matriz cmcut (Transposta2)

Transposta2_corte = Transposta2(:,54:625);

%% Corte dos espectros

G1_corte=G1_ABS(:,54:625);

G2_corte=G2_ABS(:,54:625);

G3_corte=G3_ABS(:,54:625);

G4_corte=G4_ABS(:,54:625);

C_corte=C_ABS(:,54:625);

%% Plotar os graficos cortados

plot(Transposta2_corte, G1_corte)

plot(Transposta2_corte, G2_corte)

plot(Transposta2_corte, G3_corte)

plot(Transposta2_corte, G4_corte)

plot(Transposta2_corte, C_corte)

%%Montando a matriz de interesse dos 5 grupos unidos

Groups= [G1_corte;G2_corte;G3_corte;G4_corte;C_corte];

plot(Transposta2_corte, Groups)

%% Pre processamento

analysis

SG 7 JANELAS 2 POL/BASELINE 2 POL

%% Transformar dataset em double e pre-processadas

Groups_pp_d=double(Groups_pp);

Groups_pp_PCA_d=double(Groups_pp_PCA)

Groups_pp_PCA = dataset(Groups_pp_PCA);

%% Plotar os graficos em double e pre processadas

plot(Transposta2_corte, Groups_pp_d)

%% PCA

analysis

PCA 1 DIA 6 PCs/ 15 dias 4 PCs/ 30 dias 3 PCs

%% KS KENNARD STONE

% Kennard-Stone para as 5 classes de barbeiro

[Train1,Val1,Test1,Group_Train1,Group_Val1,Group_Test1]=selamostks_rp(G1_corte,ones(20,1));

[Train2,Val2,Test2,Group_Train2,Group_Val2,Group_Test2]=selamostks_rp(G2_corte,ones(20,1));

[Train3,Val3,Test3,Group_Train3,Group_Val3,Group_Test3]=selamostks_rp(G3_corte,ones(20,1));

[Train4,Val4,Test4,Group_Train4,Group_Val4,Group_Test4]=selamostks_rp(G4_corte,ones(20,1));

[Train5,Val5,Test5,Group_Train5,Group_Val5,Group_Test5]=selamostks_rp(C_corte,ones(20,1));

Group_Train2 = Group_Train2+1;

Group_Test2 = Group_Test2+1;

Group_Val2 = Group_Val2+1;

Group_Train3 = Group_Train3 + 2;

Group_Val3 = Group_Val3 + 2;

Group_Test3 = Group_Test3 + 2;

Group_Train4 = Group_Train4 + 3;

Group_Val4 = Group_Val4 + 3;

Group_Test4 = Group_Test4 + 3;

Group_Train5 = Group_Train5 + 4;

Group_Val5 = Group_Val5 + 4;

Group_Test5 = Group_Test5 + 4;

Train_2= Train2+1;

Val_2= Val2+1;

Test_2= Test2+1;

Train_3= Train3+2;

Val_3= Val3+2;

Test_3= Test3+2;

Train_4= Train4+3;

Val_4= Val4+3;

Test_4= Test4+3;

Train_5= Train5+4;

Val_5= Val5+4;

Test_5= Test5+4;

Train = [Train1;Train2;Train3;Train4;Train5];

Val = [Val1;Val2;Val3;Val4;Val5];

Test = [Test1;Test2;Test3;Test4;Test5];

Group_Train = [Group_Train1;Group_Train2;Group_Train3;Group_Train4;Group_Train5];

Group_Val = [Group_Val1;Group_Val2;Group_Val3;Group_Val4;Group_Val5];

Group_Test = [Group_Test1;Group_Test2;Group_Test3;Group_Test4;Group_Test5];

%% SPA-LDA GraFICO

[l,R,Lopt,class_test,class_pred,Errors_Pred]=selecaoaps_internalvalidation(Train,Group_Train,Val,Group_Val,Test,Group_Test,6,10);

[T]=df_plot(Train,Group_Train,l,Test,Group_Test,Group_Val,Val);

%GA-LDA

[l,optlamb,fitlog]=galda_v1p1(6,80,Train,Group_Train,Val,Group_Val,Test,Group_Test,6,10);

[T]=df_plot(Train,Group_Train,l,Test,Group_Test,Group_Val,Val);

%% Classification Milano

cm=cm';

cm_GA=cm(:,l)

Train_GA=Train(:,l)

Test_GA=Test(:,l)

Val_GA=Val(:,l)

class_gui

%% Criar as matrizes separadas para o KS CALIBRATION

Train_dia1=train_KS(Groups_pp_d)

%% Criando as matrizes separadas de cada classe

G1_PP=Groups_pp_d(1:20,:);

G2_PP=Groups_pp_d(1:20,:);

G3_PP=Groups_pp_d(1:20,:);

G4_PP=Groups_pp_d(1:20,:);

C_PP=Groups_pp_d(1:20,:);

% MATRIZ DE CALIBRAÇÃO (apenas infectadas do treino)

Xcal = [Train_G1; Train_G2; Train_G3; Train_G4];

% MATRIZ PARA PREDIÇÃO (infectadas do teste)

Xpred = [Test_G1; Test_G2; Test_G3; Test_G4];

% 1) Reescala com os dados infectados para prever infectadas

[X1, X2] = optrescale(Xcal, Xpred, []);

% MATRIZ DE CONTROLE (NÃo infectadas)

Controle = [Train_C; Test_C];

% 2) Reescala comparando com as NÃo infectadas

[X1, X3] = optrescale(Xcal, Controle, []);

DDSGUI

%%REMOVER OUTLIER

linhas_a_remover = [52,];

X1_Sem = X1(:,:);

X1_Sem(linhas_a_remover,:) = [];

DDSGUI

%%REMOVER OUTLIERS

linhas_a_remover = [5,51];

X1_Sem = X1(:,:);

X1_Sem(linhas_a_remover,:) = [];

DDSGUI

%%REMOVER OUTLIERS

linhas_a_remover = [7,38,40];

Xcal_Sem = Xcal(:,:);

Xcal_Sem(linhas_a_remover,:)=[];

%% REMOVER OUTLIERS NO TEST

DDSGUI

linhas_a_remover = [1,];

X2_Sem = X2(:,:);

X2_Sem(linhas_a_remover,:) = [];

linhas_a_remover = [1,16];

X2_Sem = X2(:,:);

X2_Sem(linhas_a_remover,:) = [];

%% Detalhes do DDSGUI

O numero de PC's foram 4 para cada matriz de dias de infecção

O Pre processamento foi Centering

O alfa foi 0.05

Metodo de estimação foi o robusto
